# Seasonal and Regional Differences in Eating Times in a Representative Sample of the Brazilian Population

**DOI:** 10.3390/nu15184019

**Published:** 2023-09-16

**Authors:** Jefferson Souza Santos, Debra Jean Skene, Cibele Aparecida Crispim, Claudia Roberta de Castro Moreno

**Affiliations:** 1 Department of Health, Life Cycles and Society, School of Public Health, University of São Paulo, São Paulo 01246-904, Brazil; jeffersonsouza@usp.br; 2 Chronobiology, Faculty of Health and Medical Sciences, University of Surrey, Guildford GU2 7XH, UK; d.skene@surrey.ac.uk (D.J.S.); cibele.crispim@ufu.br (C.A.C.); 3 Chrononutrition Research Group, Faculty of Medicine, Federal University of Uberlândia, Uberlândia 38405-320, Brazil; 4 Psychology Department, Stockholm University, 114 19 Stockholm, Sweden

**Keywords:** food intake, latitude, season, region, chrononutrition

## Abstract

Human food intake and its timing are a complex behavior that can be influenced by a variety of factors, some of which may vary from season to season or from region to region. In this study, our aim was to investigate the seasonal variation in food intake times, with a particular focus on how these may vary across different regions of a country. We conducted an analysis of data from 20,622 adults from the National Household Budget Survey (POF-IBGE), encompassing complete food diaries collected from individuals residing in Brazil, and thereby ensuring representation across different latitudes. Each participant’s daily food intake was reported for two non-consecutive days at different times in the same week using food diaries. An ANOVA revealed a later food intake time in the evening in high-latitude regions compared to low-latitude regions. The Sidak post-hoc test showed a significant interaction effect between region and season, demonstrating a pattern of early First Intake Time and Eating Midpoint in the Northeast region during spring/summer. Additionally, we observed an independent effect of the region, as early food intake times were found in low-latitude regions. These findings offer a basis for discussing food intake times among individuals living in different regions located on distinct latitudes.

## 1. Introduction

Animal behavior is constantly influenced by fluctuations in resource availability, competition, and diseases, which may present a seasonal pattern [1,2]. In humans, several factors may affect food intake, including the following: economic factors, which influence individuals’ access to food depending on their income level [3,4,5]; personal preferences, such as the habit to eat healthier or to taste new foods [4]; and lifestyle changes, or new work/social schedules and routines [6]. In addition, seasonal changes, e.g., when people prefer to consume more fruits and vegetables in summer while increasing their consumption of more high-calorie foods in winter [7,8,9], may be considered the main factor contributing to the annual variation in human food intake.

Seasonality is a critical factor modulating food intake, which must be considered in epidemiological studies. The pattern of seasonal food intake seems to be influenced by climatic conditions, with more pronounced fluctuations present in temperate regions than tropical regions [10]. In addition, it has been demonstrated that the food content varies according to purchasing power, with a concentration of high purchasing power in the Southeast and South regions [11]. Moreover, food intake tends to be modulated by seasons within the same Brazilian region. In the South region, for instance, the presence of carbohydrates was reported to be higher in summer, while the total fat intake was higher in winter [12].

On a global scale, the seasonality of food intake has exhibited a general pattern where cold seasons are linked to a higher consumption of hot food and alcoholic beverages, and warm seasons are related to a higher consumption of fruits and dairy products [5]. This may vary depending on the climatic zone; for example, in West Africa regions, the food intake pattern varies with the rain season and not with the environmental temperature [5]. Regions located near the Equator present a similar pattern of one wet season followed by a dry season [5], which makes the task challenging for epidemiologists to determine a fixed seasonal food intake pattern. These surveys are important in order to understand seasonal fluctuations in food intake, but they are limited to cross-sectional data, including small sample sizes.

The National Household Budget Survey (POF) is a populational-based survey that aimed to collect data about the composition of the family budget to build consumption patterns for Brazilian families. The National Dietary Survey is a sub-survey from POF to investigate food intake at an individual level in order to assess food patterns in the Brazilian population [13]. Based on these data, the POF performs an analysis of personal food intake, generating indicators that are used to understand the profile of food intake by the Brazilian population in different age groups, geographic regions, and income levels [14].

The regional and seasonal differences in food intake have been mostly described in countries located in Europe and the USA. Considering the size of Brazil, which spans a wide range of latitudes, there has been increased interest in investigating a potential latitude/seasonal effect on food intake times in the different regions. Thus, the aim of this study was to investigate the variation in food intake times across the year according to seasons and latitudes, using a well-represented sample from the Brazilian population. 

## 2. Methods

### 2.1. National Household Budget Survey (POF-IBGE/2008–2009)

This study used the National Dietary Survey from the POF database. This sub-sample originated from the Individual Food Consumption section which collected data about food intake. This survey collects data about the quantity and types of foods, frequency and time of consumption, and, for specific food items such as meats and vegetables, the cooking method. The National Dietary Survey was performed on all individuals aged 10 years old and above in 13,569 randomly selected households, representing 24.3% of the 55,970 households screened in the POF 2008–2009. Individual food intake data from 34,003 individuals were obtained [14].

The National Dietary Survey began on 19 May 2008, and finished one year later on 18 May 2009. Food intake details were obtained in two non-consecutive days by self-reported food diaries. The two food diary collections took place within a week for each participant. The method of food intake based on self-reports has the benefit of being memory independent, since food recording was completed at the same time as eating [14].

Data from all the states of Brazil (n = 23 + Federal District) were gathered based on the sampling weights assigned to each selected sampling unit (families). It was necessary to correct possible sample deviations in relation to the population to ensure that the research results were representative of the population studied. The sampling weight criteria used in the POF considered several factors, such as the size of the sampling unit (family), the probability of selecting the sampling unit, the survey response rate (i.e., the proportion of families that effectively responded to the survey), the allocation of the sample to different geographical and social strata, and other statistical adjustments to ensure the representativeness of the sample population. Additional information about the sampling weight criteria used in the POF may be obtained in the IBGE official publication [14]. In the present study, the South and Southeast regions were considered high latitudes (from 14° S to 33° S) compared to low latitudes in the North and Northeast regions (4° N to 18° S). 

### 2.2. Sample Characteristics

The method of the sample design adopted in the POF comprised a master sample from clusters of census sectors. Each sector was stratified according to the governmental subdivision of the household’s location, geographical area (urban or rural), and income based on the 2000 Census performed by IBGE [15]. The initial sample comprised 34,003 individuals, with the present analysis including only adults aged 18 to 59 years old (n = 21,020). Therefore, a total of 12,983 children, adolescents, and individuals over 59 years old were excluded (51.5% of females). Additionally, 398 individuals with missing data regarding the completion date of their food diaries were excluded. This information was essential to determine the season when individuals completed the food diaries. Individuals who completed only one food diary (697 cases) were also included in this study. An average of the two non-consecutive days was calculated for individuals who completed both food diaries. Thus, the final sample size comprised 20,622 adults.

### 2.3. Chrononutritional Variables

Only the variables related to the time of consumption were considered in this study, which will be treated here as “chrononutritional variables”. The variables related to food timing were the following: first food intake time, last food intake time, and Eating Midpoint. Information about the date and month of the food diary completion was also extracted.

The seasons were defined as follows: 20 March to 22 September (fall/winter) and 23 September to 19 March (spring/summer), as the data were from the South Hemisphere. The First Intake Time was determined when it occurred from 05:00 h onwards and the Last Intake Time was considered up to 04:00 h. The Eating Midpoint was defined as the midpoint time between the First and Last Intake Times, based on the following calculation:

$$\mathrm{Eating}\;\mathrm{Midpoint}=\left(\frac{\mathrm{Last}\;\mathrm{Intake}\;\mathrm{Time}-\mathrm{First}\;\mathrm{Intake}\;\mathrm{Time}}{2}\right)+\mathrm{First}\;\mathrm{Intake}\;\mathrm{Time}$$

As an example, an individual who ate the first food intake at 07:00 h and the last food intake at 20:00 h has a 13-h eating window; thus, the Eating Midpoint is at 13:30 h.

### 2.4. Statistical Analysis

The descriptive analyses (means and confidence intervals) were calculated for both sociodemographic and chrononutritional variables. The inference statistics included a Chi-squared test to investigate potential differences in frequencies to categorical variables, and two-way ANOVA to examine the effects of region (with low or high latitudes) and season on chrononutritional variables. The post-hoc comparisons using the Sidak procedure were performed on all significant interactions identified by two-way ANOVA. Both the descriptive and inferential analyses were performed taking into account the complex design of the sample. This procedure was implemented for all data analysis using either “svy” Stata commands (Stata 16 software) or the “survey” package in R (R 4.2.1) to consider the complex sample design. The significance level adopted in the analyses was set at *p* < 0.05.

## 3. Results

Table 1 illustrates a similar distribution of sex and age with equivalent proportions among categories. About 43% of the sample had more than 11 years of education. A large part of the sample identified themselves as white (49%) or black/brown (49.7%). Just over half of the sample was below the overweight line (BMI < 24.9 kg/m^2^) and 14% were obese (BMI > 30.0 kg/m^2^).

Most sociodemographic variables did not differ between the seasons within each region, with differences only being observed in the Midwest region. In this case, only age and BMI variables differed statistically between the seasons.

**Figure 1:**
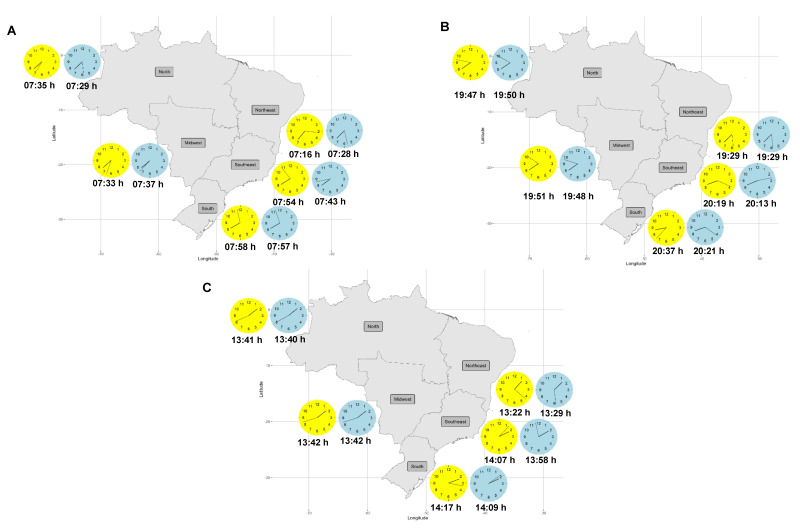
First food intake time (**A**), last food intake time (**B**), and Eating Midpoint (**C**) according to regions and seasons of Brazil.

In Table S1, the mean values of the chrononutritional variables for each region are shown by groups of seasons: spring/summer and fall/winter. The first food intake time was earlier in spring/summer (07:16 h) compared to fall/winter (07:28 h) in the Northeast region (*p* < 0.001). By contrast, this same variable was later in spring/summer (07:54 h) compared to fall/winter (07:43 h) in the Southeast region (*p* = 0.03). The last food intake time was later in spring/summer (20:37 h) compared to fall/winter (20:21 h) in the South region (*p* = 0.01). The Eating Midpoint was earlier in spring/summer (13:22 h) compared to fall/winter (13:29 h) in the Northeast region (*p* = 0.03), and was later both in the Southeast (spring/summer—14:07 h; fall/winter—13:58 h; *p* = 0.03) and South regions (spring/summer—14:17 h; fall/winter—14:09 h; *p* = 0.04). To provide a clearer visualization of the data, the means of the first and last food intake times, and the Eating Midpoint are represented in Figure 1A–C.

The yellow clock represents the chrononutritional variable during spring/summer. The blue clock represents the chrononutritional variable during fall/winter. Daylight Saving Time (DST) was introduced in three regions during the data collection period. Subsequently, an analysis was conducted to assess the impact of DST implementation on chrononutritional variables (Table S2). The results revealed no statistically significant differences in chrononutritional variables between the periods before and after DST implementation.

Two-way ANOVA was used to investigate the possible interaction between region and season for the chrononutritional variables (Table 2). It was found that both the first food intake time and Eating Midpoint showed a significant interaction between region and season. Additionally, an independent effect of the region was identified in all variables. No effect of the season was identified in any of the studied chrononutritional variables. Additional information about the means and standard deviation (SD) of the chrononutritional variables may be visualized in Table S3.

The Sidak post-hoc test was used for a better investigation of the interaction between region and season identified in the chrononutritional variables. This test allowed multiple comparisons between all regions and seasons to be performed (Figures S1 and S2). It was found that, during spring/summer, the first food intake time differed in the Northeast region compared to all other regions. The first food intake time in the Northeast region during spring/summer occurred earlier (07:16 h) compared to all other regions, with the latest first food intake time occurring in the South region (07:58 h) in the same season (Table S1).

According to the post-hoc tests of multiple comparisons, region and thus latitude have a stronger influence on the first food intake time than longitude (Figure S1). The North and Northeast regions showed significant differences between spring/summer and fall/winter when compared to the Southeast (North: 0.001 < *p* < 1.00; Northeast: 0.001 < *p* < 0.05) and South (North: 0.001 < *p* < 0.007; Northeast: *p* = 0.001) regions. The midwest region presented mixed results when compared to the seasons in the North, Northeast, Southeast, and South regions.

Another post-hoc analysis was performed on the Eating Midpoint (Figure S2). During spring/summer, the Northeast region showed significant differences compared to all the other regions and seasons. However, this difference disappeared when spring/summer was compared to fall/winter in the same region. Similar to the findings of first food intake time, the Eating Midpoint in the Northeast region during spring/summer was the earliest of all regions and seasons (13:22 h). For comparison purposes, the latest Eating Midpoint was found in the South region (14:17 h), which represents a difference of almost 60 min between these regions in the spring/summer seasons (Table S1).

According to the post-hoc analysis performed on both the first food intake time and the Eating Midpoint, there were significant differences between the Southeast/South and North, Northeast, and Midwest regions in both groups of seasons (spring/summer and fall/winter). Related to the first food intake time, the effect of latitude becomes evident when comparing the means recorded in each region. In regions with lower latitudes (North, Northeast, and Midwest), the Eating Midpoint was concentrated between 13:22 h and 13:42 h, whilst in regions with higher latitudes (Southeast and South), this variable was later (13:58 h to 14:17 h).

## 4. Discussion

This is the first study including a representative sample to evaluate seasonal variation in chrononutritional variables. Our results indicate a later food intake time as the latitude increases in Brazil. Notably, this effect appears to be more pronounced in the first food intake time compared to the last food intake time, resulting in a later Eating Midpoint at higher latitudes across the country. The latitude effect becomes stronger using multiple comparisons, showing significant differences in low-latitude regions (North/Northeast) compared to high-latitude regions (South/Southeast), reinforcing the role of latitude/regionality in determining the time of eating. Despite the absence of an independent effect of season, the data presented here indicate a significant interaction between region and season in the first food intake time and the Eating Midpoint. This finding suggests that season is not enough to influence chrononutritional factors per se, but it does have the potential to modulate food intake time when considered together with the regions of the country.

Although there is a lack of studies about regional differences in food intake time, some considerations must be pointed out about the variation of 24 h rhythms across latitudes [16]. An individual’s chronotype may be influenced by characteristics of the light/dark cycle, which varies across latitudes [17]. Thus, there is a latitudinal cline of chronotype in Brazil, considering the geographic distribution of the regions. The difference in daylight exposure across the regions produces a morning to evening direction of chronotype with the increase in latitude [17]. Accordingly, we hypothesize that evening-type individuals are more frequent in the Southeast/South regions due to being located in the highest latitudes of the country. This phenomenon helps us to understand the reason for delayed food intake times in these regions since late chronotype/eveningness has been related to later food intake times [18,19].

It is also important to highlight that the development of global trade has increased the year-round availability of previously seasonal foods [20], which appears to mitigate the impact of seasonality on the food consumption of various populations. However, little is known about the seasonal impact on meal timings. In Brazil, a previous cross-sectional study performed in a Southeast state did not identify seasonal differences in food intake time, although macro and micronutrients were different across seasons [21]. The intake of saturated, monounsaturated, and polyunsaturated fats was higher during winter among women [21]. Furthermore, Rossato and colleagues identified a seasonal effect of nutrient intake in a Brazilian South state sample [12]. Higher carbohydrate intake was linked to summer, while the largest amount of total fat ingestion was recorded in winter. These data may indicate the potential role of food energy content in modulating chrononutritional variables. The propensity to consume more calorie-dense nutrients in winter could delay the circadian clock timing system [22,23,24,25], impacting the first and last food intake times during winter. Despite these findings, it was not possible to find this pattern in our study. Conversely, early food intake times were found in fall/winter compared to spring/summer in high-latitude regions. Therefore, other factors (e.g., photoperiod) could explain the delayed food intake time during spring/summer in these regions in Brazil.

The photoperiod may influence the food intake times at higher latitudes in Brazil [26]. Regions far from the Equator commonly exhibit well-defined seasons during the year, including a marked variation in the photoperiod [16,27]. Since the days are longer during summer, this may increase the potential to delay food intake times [28]. Furthermore, physical activity becomes more frequent during the summer months [29,30,31]. These activities tend to be concentrated during the daylight hours, but when they occur in the early hours of the evening, they may delay circadian rhythms [32,33], including food intake times [34,35].

In addition, the greatest Latin American metropolises (e.g., São Paulo) are located in the Southeast region of Brazil, which allows many options for social activities during summertime. These activities encourage the behavior of outdoor gatherings, which exposes people to additional hours of daylight [36]. Together with the increase in social activities, the change in the characteristics of the meals should also be highlighted. Most people in summer prefer lighter and cold foods during the daylight hours, restricting the evening hours to high glycemic caloric snacks [12,37]. Together, these events likely contribute to delaying circadian rhythms and food intake times.

Daylight saving time (DST) must also be considered as a crucial factor in modulating food intake times. In Brazil, DST was implemented during the period of this survey (19 October 2008 to 15 February 2009) when the clocks moved forward by one hour, extending social activities into the daylight evening. Only the Midwest, Southeast, and South regions adopted DST during this period. Despite the unbalanced sample included in the analysis, there were no differences in food intake time during DST compared to conventional hours (one month before DST compared to one month after DST). Therefore, DST does not seem to affect food intake time in this representative sample.

Knowledge about the seasonal variation in food intake time for the Brazilian population will provide huge benefits to public health. The employment of nutritional planning with targeting interventions or policies could encourage the population to have healthy food intake times in a particular region or season. Additionally, chrononutrition knowledge might help health professionals incorporate it into the treatment of seasonal circadian disruptions in high-latitude regions (e.g., during the summer months when there are longer days and shorter nights, or the opposite in winter, both with the potential to trigger circadian rhythm disruptions) [38,39].

This study has some limitations that need to be stated. First, the sample was from 2008 to 2009, which may lead us to outdated interpretations. Second, the cross-sectional design of this study restricts the interpretation of the relationships among variables. Longitudinal studies comparing the same individuals across seasons from all regions of Brazil will provide a feasible scenario about both season and regional effects in chrononutritional variables. Lastly, the nature of the temporal series has meant having to use a more sophisticated analysis to investigate seasonality in this study. The 1-year temporal series of the POF (2008–2009) may not be able to get sufficient data points within each season for an accurate analysis of seasonality, since seasonal patterns require multiple observations to be robustly detected and modeled [40].

## 5. Conclusions

This is the first study to evaluate food intake times in a cross-sectional representative sample which allowed us to observe a latitudinal pattern of food intake timing in Brazil. Our results highlighted early first and last food intake times, and the Eating Midpoint in low-latitude compared to high-latitude regions of Brazil. In addition, an interaction effect was found between region and season, which also underlined the early food intake time in the spring/summer seasons in the North/Northeast regions compared to the South/Southeast regions. These findings provide a good foundation on which to base discussions about healthy food intake times, together with the quality and quantity of food intake among individuals, particularly in adult populations living in high latitudes.

## Figures and Tables

**Table 1:** Frequencies (%) of sociodemographic variables according to regions and seasons of Brazil.

	Brazil	North (n = 3003)		Northeast (n = 7504)		Midwest (n = 2963)		Southeast (n = 4545)		South (n = 2607)	
		Spring-Summer	Fall-Winter	Spring-Summer	Fall-Winter	Spring-Summer	Fall-Winter	Spring-Summer	Fall-Winter	Spring-Summer	Fall-Winter
Sociodemographic Variables ^#^	%	%	%	%	%	%	%	%	%	%	%
	(95% CI)	(95% CI)	(95% CI)	(95% CI)	(95% CI)	(95% CI)	(95% CI)	(95% CI)	(95% CI)	(95% CI)	(95% CI)
Sex											
Male	49.8	26.5	25.4	23.2	26.3	21.7	28.5	23.6	26.3	24.7	24.3
	(49.0–50.6)	(23.7–29.6)	(22.5–28.5)	(21.3–25.2)	(24.4–28.3)	(19.1–24.5)	(25.5–31.7)	(21.3–26.1)	(24.1–28.8)	(22.1–27.5)	(21.7–27.1)
Female	50.2	23.9	24.2	23.9	26.5	20.9	28.9	23.5	26.5	25.3	25.6
	(49.4–50.9)	(21.4–26.6)	(21.5–27.1)	(22.1–25.9)	(24.7–28.5)	(18.5–23.5)	(26.1–32.0)	(21.2–26.0)	(24.2–28.9)	(22.7–28.2)	(22.9–28.5)
Age											
18–25	21.8	13.8	11.8	11	12.5	8.2 *	15.1 *	10.2	10.6	9.8	10.1
	(20.9–22.8)	(11.8–16.1)	(10.0–13.9)	(9.8–12.3)	(11.3–13.9)	(6.8–9.9)	(12.6–18.1)	(8.5–12.1)	(9.2–12.2)	(8.3–11.7)	(8.4–12.1)
26–35	27.3	14.8	14	14	15.6	12.8 *	15.8 *	12.1	14	12.3	12.4
	(26.2–28.4)	(12.6–17.4)	(11.9–16.5)	(12.5–15.7)	(13.8–17.6)	(10.8–15.1)	(13.6–18.3)	(10.4–13.9)	(12.2–16.0)	(10.4–14.6)	(10.4–14.7)
36–45	24.7	10.8	13.1	10.9	12.4	10.7 *	13.4 *	11.8	13.5	11.9	14.6
	(23.7–25.7)	(8.9–13.0)	(11.1–15.3)	(9.8–12.1)	(11.1–13.7)	(8.9–12.8)	(11.6–15.5)	(10.3–13.5)	(11.8–15.4)	(10.0–14.2)	(12.4–17.1)
45–59	26.2	11	10.7	11.3	12.4	10.9 *	13.1 *	13.1	14.8	15.9	12.9
	(25.1–27.3)	(9.2–13.2)	(8.9–12.7)	(10.1–12.6)	(11.1–13.7)	(9.1–13.0)	(11.1–15.3)	(11.4–15.1)	(13.0–16.8)	(13.5–18.6)	(11.1–15.0)
Years of education											
0–10	56.9	30.9	29.8	32.3	34.5	22.8	31.6	25.3	26.5	26.2	27.5
	(55.3–58.5)	(27.0–35.2)	(26.2–33.6)	(29.7–35.1)	(31.8–37.3)	(19.7–26.1)	(28.0–35.4)	(22.5–28.4)	(23.7–29.6)	(23.0–29.6)	(24.3–30.9)
>11	43.1	19.5	19.8	14.8	18.3	19.8	25.8	21.8	26.3	23.9	22.5
	(41.5–44.7)	(16.7–22.5)	(17.0–23.0)	(13.2–16.6)	(15.9–21.1)	(17.0–22.9)	(22.0–30.1)	(19.1–24.8)	(23.4–29.6)	(20.7–27.4)	(19.6–25.7)
Race											
White	49	10.8	10.5	13.2	13.6	19.2	23.2	27.4	30.5	39.3	39.5
	(47.4–50.7)	(9.0–13.1)	(8.7–12.8)	(11.7–14.8)	(12.2–15.1)	(16.3–22.5)	(20.0–26.8)	(24.4–30.6)	(27.3–33.9)	(35.0–43.7)	(35.2–44.0)
Black/Brown	49.7	38.5	37.3	33.5	38.6	23	33.4	19.3	21.7	10.2	9.7
	(47.4–50.7)	(34.1–43.2)	(33.0–41.7)	(30.7–36.3)	(35.7–41.6)	(20.0–26.2)	(29.7–37.4)	(16.9–22.0)	(19.1–24.5)	(8.3–12.5)	(7.8–12.0)
Asian/Indigenous	1	0.9	1.6	0.4	0.5	0.2	0.6	0.3	0.5	0.5	0.5
	(0.7–1.2)	(0.4–2.0)	(1.0–2.5)	(0.2–0.7)	(0.3–1.0)	(0.1–0.5)	(0.3–1.3)	(0.1–0.9)	(0.3–0.8)	(0.2–1.2)	(0.2–1.0)
Do not know	0.3	0.2	0.1	0.1	0.2	0.2	0.2	0.1	0.2	0	0.3
	(0.2–0.5)	(0.0–0.5)	(0.1–0.4)	(0.1–0.4)	(0.1–0.3)	(0.0–0.6)	(0.0–0.6)	(0.0–0.7)	(0.0–0.6)	(0.0–0.1)	(0.1–1.4)
BMI (kg/m^2^)											
<24.9	53.1	28.4	27.1	27.8	30.6	22.7 *	33.2 *	24.5	26.5	23.1	24.5
	(51.9–54.3)	(25.0–32.1)	(24.0–30.5)	(25.5–30.2)	(28.3–33.0)	(19.9–25.8)	(29.6–37.0)	(21.9–27.4)	(24.0–29.2)	(20.4–26.2)	(21.7–27.6)
25–29.9	32.9	16.5	15.1	13.7	15.8	14.7 *	16.2 *	15.8	18.9	18.7	17
	(31.8–34.0)	(14.2–19.0)	(12.9–17.5)	(12.4–15.1)	(14.2–17.5)	(12.5–17.1)	(14.1–18.6)	(13.9–18.0)	(16.9–21.0)	(16.3–21.3)	(14.8–19.5)
>30	14	5.5	7.4	5.7	6.5	5.1 *	8.0 *	6.8	7.5	8.2	8.4
	(13.2–14.8)	(4.5–6.8)	(5.9–9.2)	(4.8–6.6)	(5.7–7.4)	(4.2–6.3)	(6.6–9.7)	(5.7–8.1)	(6.3–8.8)	(6.8–9.9)	(7.0–10.1)

National Household Budget Survey (POF/IBGE 2008-2009). Fall/winter: from 20 March to 22 September; spring/summer: from 23 September to 19 March. * Chi-squared test: *p* < 0.05 in bold. ^#^ First line shows the frequency (%) and the confidence interval is shown below it.

**Table 2:** Chrononutritional variables according to regions and seasons of Brazil.

	Regions					Seasons		Effects/Interaction		
	North	Northeast	Midwest	Southeast	South	Spring-Summer	Fall-Winter	Region	Season	Region x Season
Chrononutritional Variables	Mean (95% CI)	Mean (95% CI)	Mean (95% CI)	Mean (95% CI)	Mean (95% CI)	Mean (95% CI)	Mean (95% CI)	F *p*-Value	F *p*-Value	F *p*-Value
First Food Intake Time	07:32 (07:27–07:37)	07:23 (07:19–07:26)	07:35 (07:29–07:42)	07:48 (07:43–07:54)	07:57 (07:51–08:02)	07:42 (07:38–07:46)	07:39 (07:36–07:43)	38.71 <0.001 *	0.06 0.81	5.11 <0.001 *
Last Food Intake Time	19:49 (19:43–19:54)	19:29 (19:25–19:33)	19:50 (19:43–19:57)	20:16 (20:10–20:22)	20:29 (20:23–20:35)	20:04 (19:59–20:09)	19:59 (19:55–20:03)	77.80 <0.001 *	2.77 0.09	1.45 0.21
Eating Midpoint	13:40 (13:36–13:45)	13:26 (13:23–13:28)	13:42 (13:38–13:47)	14:02 (13:58–14:06)	14:13 (14:09–14:17)	13:53 (13:49–13:57)	13:49 (13:46–13:52)	102.76 <0.001 *	1.84 0.17	3.17 0.01 *

* Two-way ANOVA (region/season). Statistical significance (*p* < 0.05) in bold. Values are in clock time (h: min).

## Data Availability

Available upon request to the corresponding author.

## Supplementary Materials

The following supporting information can be downloaded at: https://www.mdpi.com/article/10.3390/nu15184019/s1, Figure S1: Post-hoc analysis of first food intake time; Figure S2: Post-hoc analysis of eating midpoint; Table S1: Chrononutritional variables (mean and 95% CI) according to regions and seasons of Brazil; Table S2: Mean comparisons before and after DST according to regions of Brazil; Table S3: Means and standard deviation (SD) of the chrononutritional variables according to regions and seasons of Brazil.
