# Supplementary material for: Seasonal and Regional Differences in Eating Times in a Representative Sample of the Brazilian Population
Source: Nutrients. 2023 Sep 16;15(18):4019. doi: 10.3390/nu15184019 (PMC10535183; doi:10.3390/nu15184019)
Supplement: Supplementary file 1 [file nutrients-15-04019-s001.zip › Santosetal_FigureS2.pdf]

Figure S2 – Post-hoc analysis of eating midpoint

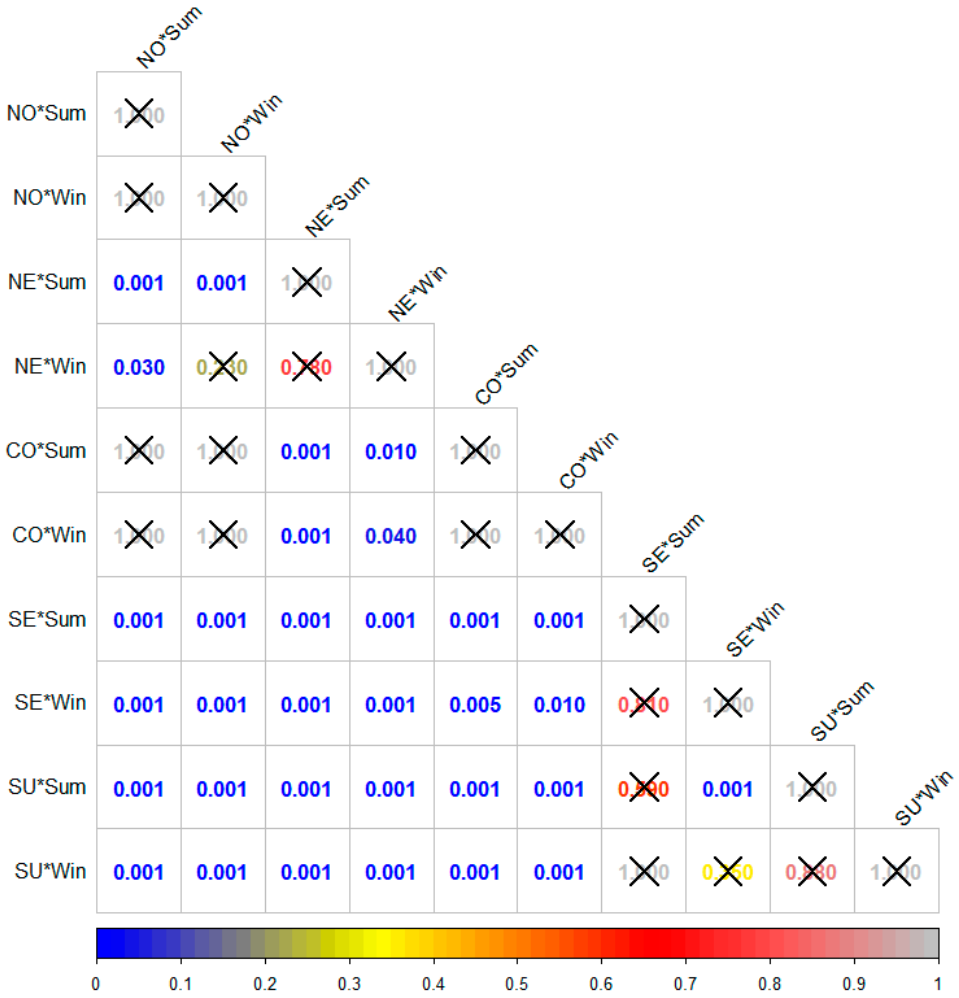

The p-values are described in each square. NO: North region; NE: Northeast region; CO: Midwest region; SE: Southeast region; SU: South region; Sum: Spring/Summer; Win: Fall/Winter.
