# Supplementary material for: Seasonal and Regional Differences in Eating Times in a Representative Sample of the Brazilian Population
Source: Nutrients. 2023 Sep 16;15(18):4019. doi: 10.3390/nu15184019 (PMC10535183; doi:10.3390/nu15184019)
Supplement: Supplementary file 1 [file nutrients-15-04019-s001.zip › Santosetal_TableS1.pdf]

Table S1 – Chrononutritional variables (mean and 95% CI) according to regions and seasons of Brazil.

	Brazil	North (n=3,003)		Northeast (n=7,504)		Midwest (n=2,963)		Southeast (n=4,545)		South (n=2,607)	
		Spring-Summer	Fall-Winter	Spring-Summer	Fall-Winter	Spring-Summer	Fall-Winter	Spring-Summer	Fall-Winter	Spring-Summer	Fall-Winter
**Chrononutritional variables**	Mean (95% CI)	Mean (95% CI)	Mean (95% CI)	Mean (95% CI)	Mean (95% CI)	Mean (95% CI)	Mean (95% CI)	Mean (95% CI)	Mean (95% CI)	Mean (95% CI)	Mean (95% CI)
First food intake time	07:41 (07:38-07:43)	07:35 (07:28-07:43)	07:29 (07:22-07:37)	**07:16* (07:12-07:20)**	**07:28* (07:24-07:33)**	07:33 (07:25-07:41)	07:37 (07:27-07:46)	**07:54* (07:46-08:03)**	**07:43* (07:35-07:50)**	07:58 (07:50-08:05)	07:57 (07:49-08:04)
Last food intake time	20:01 (19:58-20:05)	19:47 (19:39-19:55)	19:50 (19:41-19:59)	19:29 (19:23-19:35)	19:29 (19:23-19:35)	19:51 (19:42-20:01)	19:48 (19:38-19:58)	20:19 (20:09-20:29)	20:13 (20:06-20:21)	**20:37* (20:28-20:45)**	**20:21* (20:11-20:30)**
Eating midpoint	13:51 (13:49-13:53)	13:41* (13:35-13:47)	13:40* (13:33-13:46)	**13:22* (13:18-13:26)**	**13:29* (13:24-13:33)**	13:42 (13:36-13:49)	13:42 (13:35-13:49)	**14:07* (14:00-14:13)**	**13:58* (13:53-14:03)**	**14:17* (14:11-14:23)**	**14:09* (14:02-14:15)**

Means are described in clock hours (hh:min).
