# Supplementary material for: Seasonal and Regional Differences in Eating Times in a Representative Sample of the Brazilian Population
Source: Nutrients. 2023 Sep 16;15(18):4019. doi: 10.3390/nu15184019 (PMC10535183; doi:10.3390/nu15184019)
Supplement: Supplementary file 1 [file nutrients-15-04019-s001.zip › Santosetal_TableS2.pdf]

Table S2 – Mean comparisons before and after DST according to regions of Brazil.

	First food intake time		Last food intake time		Eating midpoint	
Region	Before DST (Sep 19, 2008)	After DST (Nov 19, 2008)	Before DST (Sep 19, 2008)	After DST (Nov 19, 2008)	Before DST (Sep 19, 2008)	After DST (Nov 19, 2008)
South/Southeast/Midwest	08:03 (06:30-09:35)	07:41 (07:07-08:15)	20:26 (19:27-21:24)	19:26 (18:33-20:19)	14:14 (12:59-15:29)	13:34 (13:07-13:59)
North/Northeast	07:25 (06:23-08:28)	07:02 (06:21-07:44)	19:13 (18:38-19:49)	18:04 (16:41-19:27)	13:19 (12:59-13:40)	12:33 (11:36-13:31)

The initiation of Daylight Saving Time (DST) only took place in the South/Southeast/Midwest regions on October 19, 2008. The data presented here are reported as means with corresponding 95% confidence intervals (CI). Statistical analysis comparing the means of chrononutritional variables before and after DST revealed no significant differences.
