# Supplementary material for: Seasonal and Regional Differences in Eating Times in a Representative Sample of the Brazilian Population
Source: Nutrients. 2023 Sep 16;15(18):4019. doi: 10.3390/nu15184019 (PMC10535183; doi:10.3390/nu15184019)
Supplement: Supplementary file 1 [file nutrients-15-04019-s001.zip › Santosetal_TableS3.pdf]

Table S3 – Means and standard deviation (SD) of the chrononutritional variables according to regions and seasons of Brazil.

	Regions					Seasons	
	North	Northeast	Midwest	Southeast	South	Spring-Summer	Fall-Winter
**Chrononutritional variables**	Mean ± SD	Mean ± SD	Mean ± SD	Mean ± SD	Mean ± SD	Mean ± SD	Mean ± SD
First Food Intake Time	07:32 ± 01:48	07:23 ± 01:22	07:35 ± 01:57	07:48 ± 01:02	07:57 ± 01:20	07:42 ± 01:24	07:39 ± 01:22
Last Food Intake Time	19:49 ± 02:15	19:29 ± 01:56	19:50 ± 02:26	20:16 ± 01:13	20:29 ± 01:27	20:04 ± 01:43	19:59 ± 01:42
Eating Midpoint	13:40 ± 01:34	13:26 ± 01:15	13:42 ± 01:38	14:02 ± 00:50	14:13 ± 01:03	13:53 ± 01:12	13:49 ± 01:09

Values are in clock time (hh:min)
